# Dupuytren's contracture: a retrospective database analysis to determine hospitalizations in the Netherlands

**DOI:** 10.1186/1756-0500-4-402

**Published:** 2011-10-12

**Authors:** Jetty A Overbeek, Fernie JA Penning-van Beest, Edith M Heintjes, Robert A Gerber, Joseph C Cappelleri, Steven ER Hovius, Ron MC Herings

**Affiliations:** 1 PHARMO Institute, Utrecht, the Netherlands; 2 Pfizer Inc., New London, USA; 3 Department of Plastic and Reconstructive Surgery, Erasmus Medical Centre, Rotterdam, the Netherlands; 4 Department of Health Policy & Management, Erasmus Medical Centre, Rotterdam, the Netherlands

## Abstract

**Background:**

Dupuytren's contracture is a condition of the palmar fascia involving contractures of the fascia and skin in the hand. Current treatment for Dupuytren's contracture is mainly limited to surgery. In the Netherlands, little is known about the prevalence of Dupuytren's contracture. In this study we determined the prevalence of patients with a hospitalization for Dupuytren's contracture in the Netherlands and characterized their (re)hospitalizations.

**Methods:**

From the PHARMO database, which consists of multiple observational databases linked on a patient level, all patients hospitalized for Dupuytren's contracture between 2004 and 2007 were included in the source population (ICD-9-CM code 728.6). Numbers from this source population were used to provide estimates of hospitalizations for Dupuytren's contracture in the Netherlands. Patients with a medical history in the PHARMO database of at least 12 months before their hospitalization were included in the study cohort and followed until end of data collection, death, or end of study period, whichever occurred first. Type of admission, length of stay, recorded procedures, treating specialty, number of rehospitalizations for Dupuytren's contracture, and time to first rehospitalization were assessed.

**Results:**

Of 3, 126 patients included in the source population, 3, 040 were included in the study population. The overall prevalence of patients with a hospitalization for Dupuytren's contracture was 0.04%, with the highest prevalence (0.25%) among 60-79 year old males. The majority (85%) of all hospitalizations were day-case admissions. Of the admitted inpatients (15%) the majority (81%) had one overnight stay in the hospital. The most common recorded procedure was fasciectomy (87%) and 78% of patients was treated by a plastic surgeon. During a median (IQR) follow-up of 2.9 (1.8-4.0) years, 523 patients were rehospitalized for Dupuytren's contracture. The median (IQR) time to first rehospitalization was 0.8 (0.4-1.9) years.

**Conclusions:**

This study is a first exploration of Dupuytren's contracture in the Netherlands based on hospitalizations, showing a prevalence of 0.25% among 60-79 year old males. Future studies should also address outpatient procedures to get a complete picture of the treatment of Dupuytren's contracture. In addition, patients not yet treated should be included to be able to estimate the prevalence of Dupuytren's contracture.

## Background

Dupuytren's contracture is a slowly progressing, usually painless condition of the palmar fascia involving contractures of the fascia and skin in the hand [1]. The diagnosis is based on the presence of fibromatous nodule formations in the palmar fascia, which slowly (i.e. over several months or even years) progress to cords leading eventually to contractures of joints in fingers. The condition most commonly affects the ring and little fingers, although any digit can be involved. The main consequence of Dupuytren's contracture is impaired function of the hand, which affects daily activities at the workplace and at home [1].

Little is known about the prevalence of Dupuytren's contracture in the Netherlands [2]. Several epidemiologic studies in other European countries mention a prevalence that varies widely from 4 to 11 percent, with highest rates in Northern European countries [3]. This geographic variability may be due to a genetic element, environmental factor, or a combination of the two [3]. Furthermore, prevalence of Dupuytren's contracture is highest among older men [4-6]. Although many studies have been performed, the exact etiology of Dupuytren's contracture remains unknown. Evidence suggests an autosomal dominant pattern of inheritance with incomplete penetrance [7]. Most commonly mentioned risk factors for Dupuytren's contracture are diabetes mellitus, epilepsy, smoking, alcohol consumption, and manual labor, however, not all studies support these findings [4-6,8,9]. Some studies also suggest that a lipid disorder may be an etiopathogenic factor for Dupuytren's contracture [10,11].

Disease progression is classified using a grading system. Grade 1 disease presents as a thickened nodule and a band in the palmar apononeurosis. Grade 2 presents as a peritendinous band, and extension of the affected finger is limited. Grade 3 presents as flexion contracture [1,11]. Grade 1 disease initially can be managed expectantly, but injecting the nodule with a steroid can be helpful. Surgery is recommended if function is impaired, contracture is progressing, or severe deformity is disabling. Surgery includes removing (-ectomy) or releasing (-otomy) the fibrotic cord to allow extension of the affected finger(s) and restoration of hand function. Surgical techniques available include fasciotomy, fasciectomy (radical or partial), dermatofasciectomy, and percutaneous needle fasciotomy or aponeurotomy [12]. Two studies report a proportion of 7% of patients with Dupuytren's contracture that received surgical treatment; no information was available on type of surgical treatment [4,5]. After surgery, most patients can expect a significant improvement in hand function [11]. However, surgery does not cure the disease and recurrences are commonly observed [13]. Recurrence rates are dependent on the applied surgical procedure, ranging from 8% after 6 years for dermatofasciectomy to 65% after 32 months for percutaneous needle fasciotomy [12].

Because of lacking data on Dupuytren's contracture in the Netherlands, the aim of this study was to determine prevalence of patients with a hospitalization for Dupuytren's contracture in the Netherlands and to characterize their (re)hospitalizations. This study will include more severe cases, because surgery (i.e. hospitalizations) is only recommended in the more severe cases of Dupuytren's contracture.

## Methods

### Setting

Data for this retrospective cohort study were obtained from the PHARMO Record Linkage System (PHARMO RLS), a population-based patient centric data tracking system including high quality and complete information linked on a patient level of, among other things, patient demographics, drug dispensing records from community pharmacies and hospital discharge records of approximately 2.5 million individuals from 1998 and still ongoing in defined areas throughout the Netherlands. The hospital records are obtained from the Dutch National Medical Register (LMR) [14], which comprises all hospital admissions in the Netherlands, i.e. admissions for more than 24 hours and admissions for less than 24 hours for which a bed is required. These records include detailed information concerning the primary diagnosis, procedures, and dates of hospital admission and discharge. All diagnoses are coded according to the International Classification of Diseases, Ninth Revision, Clinical Modification (ICD-9-CM). Procedures are coded according to the Dutch classification of procedures ("CvV - Classificatie van Verrichtingen"). No approval was required to access the databases, because the study was performed by employees of the PHARMO Institute (owner of the PHARMO RLS).

### Study patients

The source population included all patients with a primary hospital admission for Dupuytren's contracture (ICD-9-CM code 728.6) between January 1, 2004 and December 31, 2007. The date of the first primary hospital admission for Dupuytren's contracture in this study period was defined as the cohort entry date; consequently the source population included patients with a first hospitalization for Dupuytren's contracture, but also patients with previous hospitalizations for Dupuytren's contracture. Patients were included in the study cohort if they had a history of at least 12 months in PHARMO RLS before cohort entry date, in order to determine their co-morbidities and co-medication at the time of the first known hospitalization in the study period. Patients were followed from cohort entry date to end of data collection in the PHARMO RLS (i.e. the patient moves out of the PHARMO RLS catchment area), death, or end of the study period (December 31, 2008), whichever occurred first.

### Prevalence

The yearly number of patients with a hospitalization for Dupuytren's contracture in the Netherlands between January 1, 2004 and December 31, 2007 was estimated by extrapolating the numbers derived from the source population, standardized for age and gender [15]. Numbers are given per 100, 000 inhabitants, rounded off to 5, stratified by gender and age, and include 95% confidence intervals (CI).

### Characteristics

For all study patients the following characteristics were determined at cohort entry date: gender, age, and co-morbidities/co-medication based on hospitalizations and/or drug use in the year prior to cohort entry date including diabetes mellitus (ICD-9-CM code 250 and/or use of antidiabetics), epilepsy (ICD-9-CM code 345 and/or use of antiepileptics), and use of lipid modifying agents. In addition, the following characteristics of the hospitalization at cohort entry date were determined: type of admission (day-case or inpatient), length of stay, recorded type of procedures (fasciotomy ("CvV"-procedure codes 5-820.2 and/or 5-821.2), fasciectomy ("CvV"-procedure codes 5-823.5 and not 5-884.2, 5-892, 5-893, 5-895, or 5-896 (skin related procedures) during same hospitalization), and dermatofasciectomy ("CvV"-procedure code 5-823.5 and 5-884.2, 5-892, 5-893, 5-895, or 5-896 (skin related procedures) during same hospitalization)), and treating specialty.

### Rehospitalizations

Rehospitalizations for Dupuytren's contracture (primary discharge diagnosis ICD-9-CM code 728.6) were assessed in the period between cohort entry date and end of follow-up. The number of rehospitalizations was determined, as was the time to first rehospitalization and the treating specialty during the first rehospitalization.

### Statistical analysis

The 95% CI around the prevalence of patients with a hospitalization for Dupuytren's contracture (N per 100, 000) was calculated using the formula N ± 2*√N [16]. Characteristics of patients with a hospitalization for Dupuytren's contracture were presented descriptively. As the latest data on national trends in hospitalization show that lengths of hospital stays are declining, a Cochran-Armitage test for trend [17] was used to assess whether the proportion of day-case admissions changed in a specific direction over time.

Survival functions describing the proportion and 95% CI of patients without a rehospitalization for Dupuytren's contracture over time were computed using Kaplan-Meier survival analyses censoring patients who were considered lost to follow-up [18].

Data management and analyses were conducted using SAS version 9.1 within SAS Enterprise Guide version 4.0 (SAS Institute Inc., Cary, NC, USA).

## Results

The source population included 3, 126 patients with a primary hospital admission for Dupuytren's contracture between January 1, 2004 and December 31, 2007. Prevalence estimates of patients hospitalized for Dupuytren's contracture are shown in Table 1. After extrapolating the number of patients from PHARMO RLS to the entire population of Netherlands, we determined that each year about 40 patients per 100, 000 inhabitants were hospitalized for Dupuytren's contracture. The highest prevalence was among males 60-79 years of age: approximately 250 hospitalized per 100, 000 males each year.

**Table 1:** Prevalence of patients with a hospitalization for Dupuytren's contracture in the Netherlands between 2004 and 2007

	Number of patients per 100, 000 inhabitants (95% CI)			
	2004	2005	2006	2007
**Total (males + females)**	**35 (35-40)**	**45 (40-45)**	**40 (40-45)**	**40 (40-45)**
Males				
0-59 years	30 (25-30)	30 (30-35)	30 (25-35)	30 (25-35)
60-79 years	230 (205-255)	260 (235-285)	255 (230-280)	245 (220-270)
≥80 years	110 (65-155)	125 (75-175)	115 (65-160)	105 (65-140)
Females				
0-59 years	5 (5-10)	5 (5-10)	5 (5-10)	10 (5-10)
60-79 years	55 (45-65)	70 (60-85)	70 (60-85)	65 (55-80)
≥80 years	25 (15-40)	35 (15-50)	35 (20-50)	30 (15-40)

CI = confidence interval

**Figure 1:**
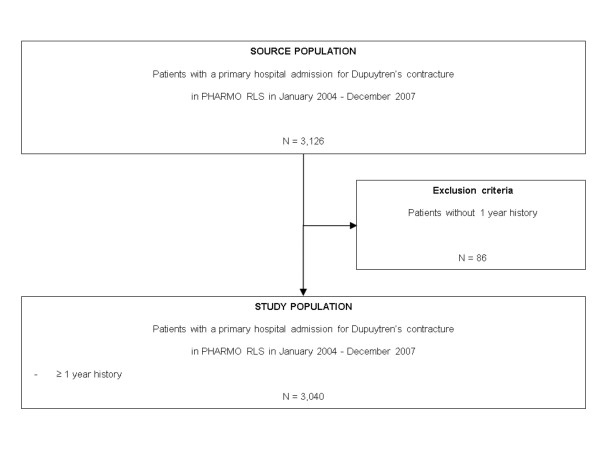
**Flowchart of selection of patients with hospitalization for Dupuytren's contracture**.

A total of 3, 040 patients (97%) had a history of at least 12 months in PHARMO RLS and were included in the study population (Figure 1). Of these patients, 76% were male and the mean (± SD) age was 62 (± 12) years (Table 2). Diabetes mellitus and epilepsy were present in 11% and 2% of the patients, respectively and 22% of the patients used lipid modifying agents.

**Table 2:** Characteristics of patients with a hospitalization for Dupuytren's contracture

	Total* N = 3, 040 n (%)
**Gender**	
Male	2, 297 (76)
Female	743 (24)
**Age (years)**	
0-19	15 (< 0.5)
20-44	191 (6)
45-59	925 (30)
60-69	1, 043 (34)
70-79	718 (24)
≥80	148 (5)
mean ± SD	62 ± 12
Co-morbidities/co-medication**	
Diabetes Mellitus	323 (11)
Epilepsy	59 (2)
Use of lipid modifying agents	683 (22)
**Type of admission**	
Day-case	2, 591 (85)
Inpatient	449 (15)
**Procedures for Dupuytren's contracture**	
Fasciotomy	32 (1)
Fasciectomy	2, 634 (87)
Dermatofasciectomy	49 (2)
Combination of fasciotomy, fasciectomy, and/or dermatofasciectomy	2 (< 0.5)
Other procedure for Dupuytren's contracture^†^	44 (1)
No procedure	279 (9)
**Surgical specialty**	
General surgeon	518 (17)
Plastic surgeon	2, 362 (78)
Orthopedic surgeon	156 (5)
Other specialty	4 (< 0.5)

SD = standard deviation; *Number of patients with a hospitalization for Dupuytren's contracture in January 1, 2004 and December 31, 2007; **Based on hospitalization(s) and/or drug use in the year prior to cohort entry date; ^†^Most observed procedures were tenotomy (n = 7), tenectomy (n = 6), and tenolyse (n = 5)

Eighty five percent (85%) of the hospitalizations were day-case admissions, this proportion increased from 82% in 2004 to 92% in 2007 (p < 0.0001). Among patients with an inpatient admission (15%, n = 449), 81% had one overnight stay in the hospital. The most common recorded procedure was fasciectomy (87%) and 78% of patients was treated by a plastic surgeon.

**Figure 2:**
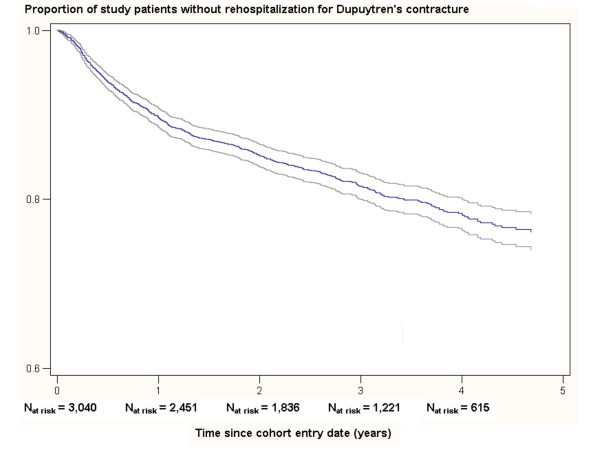
**Proportion of study patients without a rehospitalization for Dupuytren's contracture over time**. NOTE: Incomplete follow-up was censored due to loss of follow-up in the PHARMO RLS or due to death.

During a mean (± SD) follow-up of 2.9 (± 1.4) years, 523 patients had a rehospitalization for Dupuytren's contracture. After a period of 4 years, the proportion of patients without a rehospitalization for Dupuytren's contracture was 78% (95% CI: 76%-78%), resulting in 22% (95% CI: 20%-24%) of patients with a rehospitalization for Dupuytren's contracture (Figure 2). Of all patients with a rehospitalization, 85% had 1 rehospitalization (median (IQR) follow-up: 3.2 (2.1-4.2) years), 13% had 2 rehospitalizations (median (IQR) follow-up: 3.6 (2.8-4.4) years), and 2% had 3 or more rehospitalizations (median (IQR) follow-up: 4.1 (3.5-4.8) years). Overall, the median (IQR) time to first rehospitalization was 0.8 (0.4-1.9) years. The type of treating specialist during rehospitalizations was nearly always (94%) identical to that during initial hospitalization at cohort entry date.

## Discussion

In this population-based study the prevalence of patients with a hospitalization for Dupuytren's contracture in the Netherlands was estimated at 0.04%. The highest prevalence of 0.25% was among men 60-79 years of age, which is comparable to the general finding that Dupuytren's contracture is known to mainly affect middle-aged and elderly males [4-6].

Prevalence rates were based on hospitalization discharge records in the PHARMO RLS, which is representative of the Netherlands. These records include a single, mandatory primary diagnose code and may include one or more optional secondary diagnose codes. As the latter may relate to a concurrent diagnosis during hospitalization as well as to a relevant diagnosis from the past, we only selected patients with a primary diagnose code for Dupuytren's contracture. Consequently, some hospitalizations for Dupuytren's contracture were missed, but this is expected to concern only a very small number. Furthermore, it is important to realize that the observed rates of hospitalizations for Dupuytren's contracture do not represent the rates of treatment for Dupuytren's contracture, as procedures performed during an outpatient visit, presumably mostly percutaneous needle fasciotomy, were not included. Outpatient visits do not require admission and are not registered in the PHARMO database.

Our prevalence estimate was lower compared to other European studies. In the Reykjavik study, a population-based prospective cohort study [4], 19% of 1, 297 men ≥45 years of age showed signs of Dupuytren's contracture and 1.4% of them had been operated for Dupuytren's contracture. Zerajic and Finsen [5] examined hands of 610 males and 597 females ≥50 years of age in Bosnia and Herzegovina and 2.6% of males and 1.2% of females stated that they had been operated for Dupuytren's contracture. Although not clearly stated by the authors, we assume this included procedures during hospital admissions as well as outpatient procedures.

Partial fasciectomy is the most widely used procedure for the management of Dupuytren's contracture [12,19,20]. In our study, for 87% of patients hospitalized for Dupuytren's contracture a fasciectomy was recorded, which corresponds to a database study in the UK [21]. An important remark regarding this result is that, in the Netherlands, similar to the situation regarding discharge diagnoses described above, physicians are obligated to register one procedure, i.e. the primary procedure, but not necessarily all procedures performed. This would imply that the number of procedures used to manage Dupuytren's contracture may be incomplete and underestimated, but given that the extent of this misclassification is expected to be small or inconsequential, the degree of underestimation is also expected to be small or inconsequential. Furthermore, dermatofasciectomy, which includes two procedures, may have been slightly underestimated as well.

Surprisingly, for about 9% of patients no procedure was recorded during their admission for Dupuytren's contracture; unfortunately, there is no explanation for this finding.

Regarding the co-morbidities potentially increasing the risk of Dupuytren's contracture, we found that 11% of the study patients had diabetes, 2% had epilepsy, and 22% used lipid modifying agents. In the absence of an age and sex matched control group, we cannot judge whether these proportions are high. In the general Dutch population 4% has diabetes mellitus [22], 1% epilepsy [23], and 9% use lipid modifying agents [24]. However, this concerns a population with a lower mean age and a higher proportion of women, therefore it is difficult to assess whether the prevalence of these co-morbidities is indeed increased among patients with Dupuytren's contracture.

In our study the percentage of day-case versus inpatient admissions increased significantly over time, which is comparable to the increase reported by Gerber et al. [21]. The increase in our study might be explained by the growing popularity of percutaneous needle fasciotomy in the last years. This minimally invasive treatment with good short-term results is likely to be performed during a day-case admission.

After a period of 4 years, about one-fifth (22%) of the patients had a rehospitalization for Dupuytren's contracture. It was not possible to label rehospitalizations as recurrences, because we do not know whether this concerned the same hand or finger as the hospitalization at cohort entry date. Between 2004 and 2007, Gerber et al. [21] found that about 20% of the patients had two or more hospital admissions for Dupuytren's contracture within one year. The recurrence rate after fasciectomy is about 41% after 5 years [12].

This study was limited to hospitalizations for Dupuytren's contracture and should be considered as a first exploration of Dupuytren's contracture in the Netherlands. Future studies should also address outpatient procedures to get a complete picture of the treatment of Dupuytren's contracture. In addition, patients not yet treated should be included to be able to estimate the prevalence of Dupuytren's contracture.

## Conclusions

In conclusion, the prevalence of patients with a hospitalization for Dupuytren's contracture was 0.04%, with the highest prevalence (0.25%) among 60-79 year old males. About one-fifth of patients had a rehospitalization within 4 years. Estimates of the prevalence and treatment of Dupuytren's contracture in the Netherlands will be higher and should be addressed in future studies including additional data.

## Competing interests

Jetty A. Overbeek, Fernie J.A. Penning-van Beest, Edith M. Heintjes, and Ron M.C. Herings are employees of the PHARMO Institute, which received payment from Pfizer Inc in connection with the development of this study and manuscript. Robert A. Gerber and Joseph C. Cappelleri are employees of Pfizer Inc.

This study, including manuscript development, was financially supported by Pfizer Inc. The authors were allowed to conduct the study and write the manuscript independently of Pfizer's involvement (except as authors).

## Authors' contributions

All authors proposed key elements for and made significant contributions to the study design and analyses. JO, EH, RG, and JC developed the appropriate methodology for the analysis. JO, FP, EH, RG, and RH played a key role in the evaluation and assessment of the results. JO carried out the analyses and drafted the manuscript. All authors provided direction and intellectual content for the manuscript, participated in reviews, and submitted written approval of the final version.
